# Initiator or Intermediary? A Case Study on Network Relation of Environmental Regulatory Capture in China

**DOI:** 10.3390/ijerph17249152

**Published:** 2020-12-08

**Authors:** Yu Tu, Benhong Peng, Ehsan Elahi, Weiku Wu

**Affiliations:** 1 School of Economics and Management, Tsinghua University, Beijing 100084, China; wuwk@sem.tsinghua.edu.cn; 2 School of Management Science and Engineering, Nanjing University of Information Science & Technology, Nanjing 210044, China; 002426@nuist.edu.cn; 3 Binjiang College, Nanjing University of Information Science & Technology, Wuxi 214105, China; 4 Business School, Nanjing University of Information Science & Technology, Nanjing 210044, China; ehsanelahi@nuist.edu.cn

**Keywords:** environmental regulatory capture, core power circle, “core-periphery” structure, social network analysis, case study

## Abstract

Behind the environmental regulatory capture (ERC) lies a complex network of interactions and interests. Identifying the roles of stakeholders in the ERC network and their behavioral motives can illuminate the mechanism of ERC incidents, and provide policy recommendations for reducing other types of regulatory capture. Drawing on the regulatory capture and principal-agent theories, this study develops a triple-layer principal-agent model of environmental regulation practices in China. We further conduct a social network analysis (SNA) on the ERC case in the Environmental Protection Bureau (EPB) of Bobai County, Guangxi Province, China to illustrate the hidden interactions and interest transmission structure among stakeholders in ERC. The results show that the ERC presents obvious characteristics of concealment and complexity, and individual capture often evolves into collective capture. Different stakeholders are in different positions and play different roles in the network. The environmental regulatory authority, the commissioned regulatory agency, and the agency of enterprises form the core power circle of the ERC network, in which the first two play the role of intermediary and the latter acts as an initiator. They together occupy the structural hole position and dominate the evolution of the ERC network. Peripheral structure stakeholders play the role of “bridge” and profit from the expanding ERC network. It is recommended that the principle of decentralization and the balance of power must be taken into consideration. Quantitative analysis methods such as SNA should be applied to clarify accountability when punishing responsible persons. More subjects are also encouraged to participate in environmental regulations and report illegal acts actively. Finally, a blacklist system should be established in the field of environmental protection for regulating the professional and social morality of all parties.

## 1. Introduction

China has successfully transformed from one of the poorest countries in the world to the second largest economy in a 40-year period of rapid development. A positive trend in its economic growth and improvement in inhabitants’ lives has been observed [1]. However, frequent safety accidents and livelihood problems reflect a severe phenomenon of regulatory capture in China. Regulatory capture means that interest groups control regulatory policy makers or regulators in various ways (e.g., lobbying and bribery) to seek favorable regulatory policies or implementation results [2,3,4]. Regulatory capture often leads to market failure and damage to the public interest. Under such circumstances, the design and implementation of regulatory policies serve the regulated organizations. For example, the “Baby Milk” scandal shocked the whole country in 2008. The incidents of kidney stones in infants caused by poisoned milk powder were frequently exposed by the media before the situation deteriorated further, whereas due to the hidden interest transmission and regulatory capture, the local government did not take timely regulatory action after receiving public tip-offs, but deliberately concealed the facts and shielded enterprises’ illegal acts. Besides this, the phenomenon of regulatory capture also occurred in the Shuanghui meat additives incident in 2011 and the poisonous runway incident in 2016. Significantly in the expired vaccines incident in 2018, regulatory capture directly led to about 250,000 expired vaccines flowing to the market, triggering consumers’ great panic about vaccine safety in China. It can be seen that behind almost every security incident lies a serious problem of regulatory capture, which substantially threatens residents’ health and interests and leads to a critical crisis of government trust [5].

Concerning the development of ecological civilization, the Chinese government has been steadily enhancing its efforts towards environmental regulations. Chinese enterprises are faced with more and more stringent constraints during the processes of establishment and operation, including environmental impact assessment (EIA), pollution discharge supervision, and environmental law enforcement and punishment, which have all effectively curbed those illegal business activities [6]. However, the phenomenon of regulatory capture in the field of environmental protection in China is still profoundly grievous [7]. As a matter of fact, we can all find the collusion between government and businessmen in the air pollution, water pollution, garbage disposal, and other environmental problems in China.

**Figure 1:**
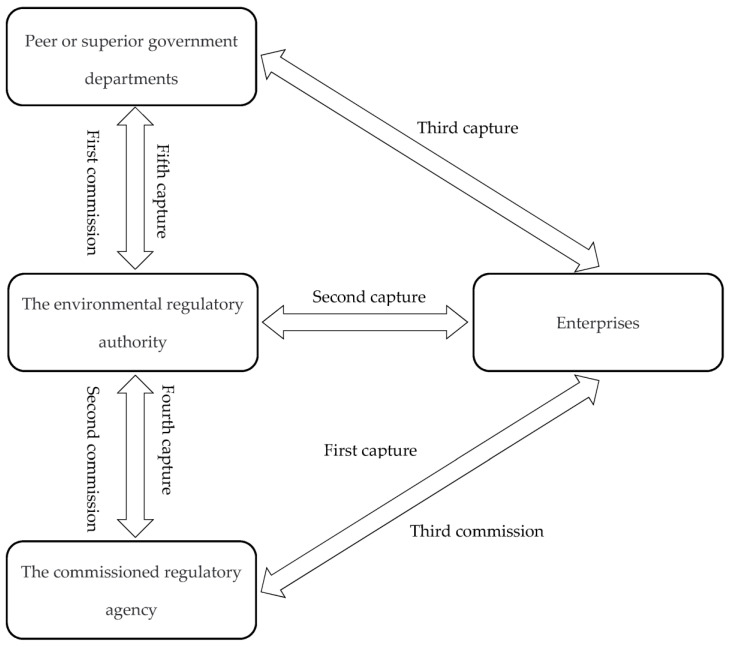
The triple-layer principal—agent model of environmental regulation practices in China.

According to the classic principal—agent model [8], the environmental regulatory authority (i.e., the principal) does not know the technologies, production, costs, and other operational information of the enterprise (i.e., the agent). As a result, it cannot accurately determine the intensity of environmental regulations. The commissioned regulatory agency (i.e., the supervisor) possesses the time, technologies, expertise, talents, and other dedicated resources to investigate the targeted enterprises in detail. Therefore, there is a first-layer principal—agent relationship in which the regulatory agency is entrusted to supervise businesses instead of the environmental regulatory authority. At the same time, the commissioned regulatory agency requires enterprises to truthfully report business information, which constitutes the second-layer principal–agent relationship. In the context of information asymmetry, enterprises are most motivated to capture the commissioned regulatory agency in order to conceal the true business information and evade regulations. Although the commissioned regulatory agency can enforce the policy impartially, it may accept the captured behavior implemented by the enterprise for its own interests, and conspire with the enterprise to deceive the environmental regulatory authority [9]. Commissioned regulatory agencies thus become the main targets for capture [10]. Of particular note, one basic assumption of the double-layer principal–agent model is that the supervisor is an independent third party. In the reality of environmental regulations in China, the commissioned regulatory agency is directly formed and managed by the environmental regulatory authority. In addition, the principal—agent model assumes that the principal is Congress as the highest organ of state power [11]. However, China’s environmental regulatory authority, i.e., the Environmental Protection Bureau (EPB), is also established and supervised by the government, essentially forming a triple-layer principal—agent relationship (Figure 1). Taken together, environmental regulation practices in China show obvious hierarchical and complex characteristics, and the resulting environmental regulation capture (ERC) involves mixed actors and their complicated relationships.

It is because there is a complex social network between actors in the case of ERC that a third party makes it difficult to distinguish the network subjects, their behavior and interactions among them. As a result, the existing research mainly discusses the institutional incentives and consequences of regulatory capture at the macrolevel [12,13], but pays less attention to the specific regulatory capture in the field of the environment. The lack of in-depth research on the behavioral interactions of all parties and the network structure underlying ERC cannot provide practical guidance for perfecting the environmental regulation system and curbing potential ERC problems [14]. Accordingly, three key ERC issues require further elucidation. First, what are the structural characteristics and core actors of the ERC network? Second, what are the components and interest relationships in each sub-network? Third, how about the strength comparison and behavior motives among stakeholders? Mainly, this study conducts a thorough analysis of the ERC case in Bobai County’s EPB of Guangxi Province, China, and is intended to explore the hidden interactions between actors at different levels. Based on the triple-layer principal—agent model, the current research can make up for the omission of complexity in ERC problems in the past literature. The role analysis of network actors (e.g., initiator and intermediary) helps to identify the mechanism of ERC and provides valuable suggestions for preventing other types of regulatory capture.

The remainder of this paper is structured as follows. We introduce the theoretical background of regulatory capture theory in Section 2, followed by a comprehensive account of relevant research in the field. Section 3 elaborates the social network analysis (SNA) method and the details of a practical case selected in this paper. Then, in Section 4, quantitative analysis results and discussions are presented around the three key issues. Finally, Section 5 draws conclusions and offers policy suggestions, and points out new ideas for studies on regulatory capture.

## 2. Literature Review

### 2.1. The Development of Regulatory Capture Theory

As a manifestation of regulatory capture, ERC is based on the regulatory capture theory Stigler [2] brought forward in 1971. Stigler (1971) [2] argues that, on the one hand, government officials are prone to regulatory capture due to the pursuit of individual interests such as wealth growth and power expansion. On the other hand, enterprises can raise the market access threshold and take over the exclusive market with the aid of regulatory capture. As a result, enterprises have the motivation to capture government behaviors in order to make regulations beneficial to their business. Therefore, the regulatory capture theory emphasizes that government regulations serve the interests of the regulated enterprises or industries. Finally, enterprises or industries can increase their profits through the redistribution mechanism of social wealth [15]. Regulatory capture theory is essentially different from the rent-seeking theory (which focuses on the active intervention of power owners) [16] and the theory of political connection (which pays attention to the close connection between the enterprise and the authority) [17]. The capture phenomenon partly stems from the pursuit of self-interest by government departments or their staff, and enterprises enhance their motivation for being captured. However, the Chicago school holds the view that the implementers of regulatory capture are not limited to enterprise groups. Peltzman (1976) [18] integrated the role of consumers and other interest groups into the analytical framework, and found that the regulatory capture behavior implemented by enterprises was uncertain, and consequences were often influenced by the interactions among different stakeholders. The introduction of stakeholder theory helps the researcher break through the perspective of a single subject, while it is still difficult to explain the mechanism of collective regulatory capture in real life because we do not understand the rights and responsibilities of different stakeholders. To this end, Laffont (1991), a representative of the Toulouse school, considered the asymmetry characteristic of information and conducted a normative study on this issue based on the principal—agent theory. The double-layer structure of “principal—supervisor—agent” that he established showed that regulators (i.e., supervisors) might conceal factual information for personal gain, or only report information beneficial to agents (i.e., enterprises) to their clients (i.e., the congress) after obtaining the real data [8]. Thus, the involvement of intermediaries improves the level of uncertainty in the cross-layer communications and interactions, providing preconditions for regulatory capture [19]. The consideration of multi-stakeholders and information asymmetry lays the basic framework for analyzing regulatory capture networks. The framework of “single regulator—single supervisor—multiple stakeholders” helps to break through limitations on individual numbers and organizational boundaries, and has a vital interpretation significance in practice. This theoretical model has also provided guidance for western countries to weaken their government regulations’ intensity in the last century [14].

### 2.2. Researches on Regulatory Capture

There is a growing body of research investigating the regulatory capture issue. Scholars mainly conduct their studies from the economic and social perspectives. In the field of economic regulations, Markusson and Haszeldine (2010) [20] studied the “capture ready” regulation in the UK capital market for fossil fuel power plants. They found that although the UK government had promised the lowest carbon emissions along with the background of global carbon emission reduction, it still could not eliminate the phenomenon of regulatory capture given the serious uncertainties in capture readiness works. Besides this, by employing a two-step approach to case studies and empirical analysis, Tudoroiu (2015) [21] suggested that enterprises would play a fierce game in capturing government leaders and influencing regulatory policies in order to suppress competitors in the market. Especially when this game process involves a huge interest group or a long industrial chain, the concerns of collective interests may exacerbate the contradictions between groups, and lead to collective regulatory capture. Indirect support for this viewpoint comes from Shahab et al.’s (2019) study on transferable development rights (TDR) programs [19]. The existence of intermediaries enhances the uncertainty and complexity in any TDR transaction, thereby increasing the transaction costs and reducing the net social benefits of the policy instruments. A streamlined policy system is therefore suggested to mitigate the considerable impact of intermediaries and to reduce the likelihood of regulatory capture. Asian scholars are equally concerned about the regulatory capture issues. Hong and Kim (2017) [22] questioned the view of “careerists’ premium” based on performance data of Korean state-owned enterprises. This study concluded that the gap between qualitative and quantitative evaluation scores increased significantly when the investigated enterprises were managed by professional managers retired from governmental agencies, thus indicating that the often-proven “careerists’ premium” did not necessarily signify the more excellent expertise of managers. However, these conclusions might also have resulted from lobbying and regulatory capture.

In the field of social regulations, scholars have conducted research on regulatory capture in environmental governance, food and drug safety, and production safety. Marsden et al. (2000) [23] took the Environmental Improvement Program by the sewage treatment industry in Tasmania as an example, and studied the case of a local government capturing federal environmental regulatory agencies. They emphasized the importance of third-party independent environmental supervision agencies in avoiding ERC in Australia. Relatedly, taking the Environmental Protection Agency (EPA) in the early Trump administration as a research object, Dillon et al. (2018) [24] used the matched data of news, official documents, and interviews from retired EPA employees to illuminate the causes of ERC. The regulated industries captured the new government’s policy guidance in terms of personnel appointments, executive orders, and budgets, and thus dismantled the EPA’s capacity to promote public health and environmental protection. Jane et al. (2015) [25] analyzed the qualification authentication of genetically modified food in Vermont, USA. They argued that food companies would interfere with food safety regulation policies by deliberately concealing negative information or fabricating false information owing to the huge information asymmetry among regulators, residents and enterprises. Perchard and Gildart (2015) [26] demonstrated the mechanism of the British Mining Association and coal mining enterprises capturing coalmine safety production regulations. It was stated that the industry association mainly enumerated the wrong idea of “coal dust was harmless to workers and the atmospheric environment” to obstruct the process of formulating occupational lung disease regulations, thus explaining the public health crisis that occurred in the British mining industry between 1918 and 1946. In addition, taking the Swedish explosive industry as a research object, Sabo and Andersson-skog (2017) [27] showed that the long-term cooperation between enterprises and regulatory authorities reduced the health and safety risks posed by explosives. At the same time, it also captured the regulatory policies, thus helping enterprises to implement unfair competition in the market. Besides, scholars have also carried out in-depth discussions on the issue of regulatory capture in view of the different situations of reforming nations and developed economies [28,29].

### 2.3. Research on Regulatory Capture in China

China, the largest developing country in the world, is in a transitional period with rapid changes in all aspects of society. The issue of regulatory capture has attracted wide attention from Chinese academia. Lu et al. (2007) [30] previously paid attention to the problem of regulatory capture in China’s economic market. From the aspects of market access qualification restriction, market scale or scope limitation, illegalness and irrationality of regulation policy, and the profit chain between taxi companies and the authorities, their analysis clarified that the regulatory capture problem did exist in the Hefei taxi market, then explained the group events among local taxi drivers. Du (2014) [31] analyzed the regulatory capture problem and governance practices in Chinese monopoly industries from the macrolevel, and further put forward the direction and path of corruption control from three perspectives, including property right reform, policy system reform and economy structure adjustment. Gong et al. (2015) [32] concentrated on this issue within food enterprises who bore the policy burdens of regional development, economic taxation, and employment. It was found that the regulatory capture incurred by policy burdens was the key reason for the absence of food safety regulation. Thus, it was suggested that the government was supposed to promote changes in the regulatory patterns, such as independent judiciary, vertical supervision and social supervision. It can be seen that regulatory capture is widespread in all walks of life, especially in vital industries involving residents’ lives (e.g., the transportation and food industries mentioned above). A sound policy system helps to reduce the occurrence of regulatory capture. Unfortunately, there will be regulatory capture in the process of establishing the policy system itself, because this process is essentially the interaction and game of different stakeholders. Stakeholders who occupy the key positions or nodes are often able to lead the construction of policies that benefit their interests.

Concerning the capture phenomenon in China’s environmental regulations, Guo and Yao (2014) [33] argued that polluting enterprises and local authorities might gradually form a community of interests. Local governments would “cover up” negative emission information for related enterprises once the strength of government–enterprise connection rose above 31.17%, which indicated a possible regulatory capture problem in local government departments. Lei and Gao (2017) [34] discussed the driving factors bringing about regulatory capture based on game theory. Their study found that the stronger the economic development desires were, the larger the scale of an enterprise was, the lower the pollution emission was, the worse the residents’ environmental protection consciousness was, and the more likely regulatory capture problems happen. Furthermore, they proved the contribution of non-governmental organizations in environmental regulations. At a more microlevel within the enterprise system, Xie (2018) [35] investigated the moderating effect of regulation capture on the relationship between environmental regulations and corporate research and development (R&D) activities. The results explained that ERC played a negative moderator role. In detail, ERC had a greater negative effect on the firms who belonged to heavy pollution industries or were situated in an area bearing lower environment protection pressure. Besides this, Shao et al. (2018) [36] built a dynamic game model between environmental regulators and polluting enterprises, laying emphasis on the possibility of regulatory capture among them, while government credibility, media disclosure, and stakeholder pressure could effectively restrain the ERC problem and curb the possibility of rent-seeking by the authority. Strict administrative penalties might help to force enterprises to conduct green production, thus reducing the opportunities for environmental regulators to be captured.

In summary, the emerging research on regulatory capture shows three shortcomings: firstly, scholars mostly elucidate the capture phenomena from the macrolevel of the state or industry. Corresponding policy recommendations thus struggle to curb various types of regulatory capture events. The lack of in-depth analysis of the specific ERC incident leads to different stakeholders’ roles and motives being neglected. Secondly, international academic research mainly focuses on the regulatory capture problem in the field of the economic market, but less attention is paid to the livelihood and welfare disciplines, thereby debasing the systematic integrity and practical significance of regulatory capture theory. Thirdly, empirical studies, theoretical analyses, and modeling simulation are the primary research methods adopted, while the specific context in each capture case itself is omitted, so it is difficult to avoid or solve other capture problems in real life. Therefore, this paper selects a typical capture case in the field of environmental regulations for analysis. In detail, we firstly define the main stakeholders in this case. Afterwards, an interaction network hidden behind the captured phenomenon is distinguished by the aids of the SNA method in quantifying linkages among stakeholders. Moreover, for the sake of providing policy recommendations for reducing or even eliminating other types of regulatory capture, the structure, types, main roles, and behavioral motives in the relationship network will be discussed in depth. Therefore, by elucidating the mechanism of ERC at depth, this study explains why this phenomenon exists widely in the practice of environmental regulations. In addition, we introduce the regulatory capture theory into the field of environmental protection and expand the research framework of regulatory capture. The integrated method of a case study and the social network method applied in this article also provide a methodology reference for related research.

## 3. Methodology and Materials

### 3.1. Social Network Analysis Method

The SNA method analyzes the structure and characteristics of a network by using the relationship data among actors. The method uses algebraic models and graph theory tools to quantify the relationship and degree, and elaborates the position and influence of each actor in the network. Stakeholders negotiate privately to obtain illegal gains in the process of capturing environmental regulations. Thus, complex and opaque relations are established, and a social network of power-money transactions is constituted [37]. Applying the SNA method to analyze the actor’s behavioral characteristics is helpful for clarifying the structure and mechanism in the capture network and making the relationships of ERC visible [38]. We apply an SNA method for the overall network analysis and the individual network analysis.

#### 3.1.1. Overall Network Analysis

Overall network analysis concentrates on the structural characteristics of a network, including network scale, network density and so on. Network scale refers to the number of all actors in the network. Network density refers to the tightness degree of linkages in the whole network. It is generally believed that the greater the network density, the greater the influence of a network on actors. The overall network analysis can be conducted using Equation (1).

(1) $$D=\frac{n}{N(N-1)}$$

where *D* denotes the network density, *N* is the number of theoretical linkages that exist in the network, and *n* is the number of direct linkages.

#### 3.1.2. Individual Network Analysis

Individual network analysis focuses on the actor’s position and role in the sub-network, that is, where the actor is in the network, what kind of power it holds, and how to influence or be subject to the overall network structure or other actors. We choose from three perspectives: centrality, cohesive subgroup and structural hole.

First, centrality is introduced to illustrate the position of each actor in the network. The centrality of the ERC network includes three indicators, i.e., degree centrality, closeness centrality, and betweenness centrality. Degree centrality refers to the number of direct linkages between an actor and other subjects. The greater the degree centrality, the greater the influence of an actor. Closeness centrality measures the distance between two actors. A high closeness centrality value implies the actor is not easily affected by other actors. Betweenness centrality examines an actor’s control ability of the interconnection of another two subjects. The greater the betweenness centrality, the more effectively an actor can gain benefits through such control. The formulas for closeness and betweenness centrality are as follows:

(2) $$C_{i}=\sum_{j}D_{ij}(i\neq j)$$

where *C_i_* denotes the closeness centrality of actor *i*, and *D_ij_* stands for the shortcut distance between actor *i* and actor *j*.

(3) $$B_{i}=\frac{\sum_{j}\sum_{k}F_{jk}(i)}{F_{jk}}(k\neq i\neq j,\begin{array}{cc} and & j<k \end{array})$$

where *B_i_* denotes the betweenness centrality of actor *i*. *F_jk_*(*i*) is the number of shortcuts between actor *j* and actor *k* through actor *i*, and *F_jk_* indicates the number of shortcuts between actor *j* and actor *k*.

Second, the cohesive subgroup is a set of closely related actors in the network. An essential feature of this set is that a subject in the central region can no longer be divided into smaller sets, while a subject in the marginal region is only closely related to the core subject [39]. This paper will pay much attention to the structural characteristics of cohesive subgroups with the aid of professional software, identifying which actors are at the core and which actors are on the periphery of the ERC network.

Third, the structural hole refers to the non-redundant relationships between two actors. Non-redundant actors are connected by the structural hole. Actors at the core play the role of “bridge”, thus controlling the links between other subjects to a large extent [40]. The measurement of the structural hole index includes four indicators: effective size, efficiency, constraint and hierarchy. Their formulas are given as:

(4) $$ES_{i}=\sum_{j}\left(1-\sum_{k}P_{ik}M_{jk}\right)(i\neq j\neq k)$$

where *ES_i_* denotes the effective size of actor *i*. *j* represents all the actors connected with actor *i*, *k* represents all the third actors except the actors *i* and *j*, and *P_jk_M_jk_* refers to the redundancy between actor *i* and *j*.

(5) $$E_{i}=\frac{ES_{i}}{S_{i}}$$

where *E_i_* denotes the efficiency of actor *i*, and *S_i_* is the actual size of this sub-network.

(6) $$C_{i}=\sum_{j}{\left(P_{ij}+\sum_{k}P_{ik}M_{jk}\right)}^{2}$$

where *C_i_* denotes the constraint of actor *i*. Actor *k* indicates the common adjacent subject of actors *i* and *j*, and *P_ij_* represents the proportion of actor *j* in all adjacent subjects of actor *i*.

(7) $$H_{i}=\frac{\sum_{j}\left(\frac{C_{ij}}{C/N}\right)\mathrm{In}(\frac{C_{ij}}{C/N})}{N\mathrm{In}(N)}$$

where *H_i_* denotes the hierarchy of actor *i*. *N* is the subject’s number in the neighborhood, and *C* is the sum of all actors’ constraints in the neighborhood.

### 3.2. Case and Stakeholder Analysis

**Figure 2:**
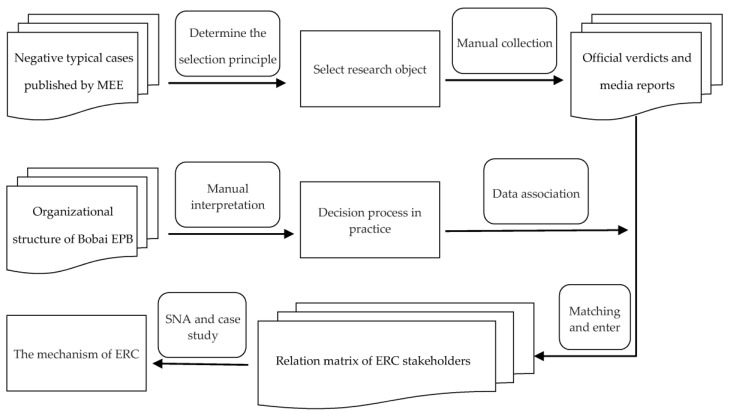
The data collection and analysis process.

Regulatory capture possesses the nature of concealment. Interaction data among stakeholders in the relationship network are difficult to obtain. For this reason, we apply a case study method to summarize the characteristics and mechanisms for relevant events with the aid of a typical ERC case to enhance the practical significance of our conclusions. Considering the public characteristic of environmental regulations, most published ERC cases are flawed in terms of information integrity. Therefore, we take the integrity of the case materials as a primary principle, followed by the typicality and credibility principles. Then, after searching 902 cases that are classified as negative and typical by the China Ministry of Ecology and Environment (MEE) from 2012 to 2015, we select the ERC case in Bobai County’s EPB, Guangxi Province, China, as the research object. The case materials are mainly from the verdict published by Yulin Intermediate People’s Court, investigation reports published by the Bobai Country Commission for Discipline Inspection, and press news from authoritative channels (e.g., SOHU.com and Yulin Daily). Multisource data ensure that the characteristics of integrity and richness, in this case, are apparent. Thus, we initially establish a relationship database of ERC. A manual interpretation of the organizational structure and work processes of the Bobai EPB is further conducted to sort out the real decision-making process and interest transmission structure in the ERC case. This complements the official investigation reports and our initial database. Finally, we establish the relation matrix of ERC stakeholders in Bobai County. The data collection and analysis process are shown in Figure 2.

From June 2011 to April 2014, Chen (hereinafter referred to as A3), the deputy chief of the Supervision Section of Bobai EPB, took advantage of his privileges to gain over CNY two million in bribes from several environmental survey consulting companies, thus ensuring the approval of EIA reports and daily operation supervision. The incident possesses typical features, such as collusion between officials and businessmen, the exchange of power and money, and collective corruption. The prominent environmental issues that concern residents are involved, such as reviewing and approving EIA reports, business production supervision, compensation for ecological damage, and public law litigation. Studies on this case are of primary importance for similar incidents in unifying adjudication standards, quantifying actors’ legal liability, and improving the environmental policy system. The characteristics of typicality and authoritativeness are also apparent. It is notable that this ERC case involves many other stakeholders in addition to A3. For ease of analysis and explanation, we first code the stakeholders involved and their units, including the authority (i.e., Bobai EPB), the supervision bureau affiliated to Bobai EPB, the evaluation unit, the agency unit, and enterprises. Then 20 representative subjects in this case are identified, and their interactions and interests are clarified, as shown in Table 1.

## 4. Results

We define that if there is an interaction or interest transmission between two stakeholders in this ERC case, their relationship is recorded as 1, otherwise it is 0. Finally, a 20 × 20 relation matrix is sorted out, and matrix nodes represent the stakeholders in the case. Then UCINET 6 and NetDraw 2 (Borgatti, S.P., Everett, M.G. and Freeman, L.C.: Harvard, MA, USA) are applied to quantify and visualize the capture network.

### 4.1. Overall Network Analysis of ERC

The number of all stakeholders in the network is the scale of this whole network. It is generally accepted that the larger the scale of a network, the more complex the structure of the network, and the greater the influence on internal stakeholders. Although there are more than 20 actual stakeholders in this case, we choose only 20 main stakeholders as research subjects because some stakeholders have the same role. For example, there are 13 EIA experts involved. Therefore, the network scale of this incident is equal to 20, and is relatively dispersed [41]. It is helpful for core stakeholders to obtain heterogeneous information and establish interest sub-networks.

UCINET 6 is applied to measure the density of this ERC network, and it is found that the network density is equal to 0.195, the average distance among internal stakeholders in this network is equal to 2.614, and the cohesion index based on the concept of “distance” is equal to 0.473. Wellman (1979) [42] reports that if the network density lies in an interval of [0, 0.25], the linkages between nodes are sparse. It is demonstrated that the main stakeholders in this ERC network are not closely connected. The whole network is loosely established and the cohesion is relatively weak. One actor’s attitude and behavior are not enough to exert a great influence on other actors. However, such a network structure, in which there are closely linked insides and sparsely connected outsides, is conducive to the formation of multiple sub-networks. This is in line with the fact that interest subjects are capable of being divided by organizational units in the actual case. It elucidates that this ERC case presents a characteristic of collective collusion, and individual capture tends to develop into collective capture. For example, D1 first implements active rent-seeking activities towards A3, while A1, A2 and A3 are in the same sub-network, and EIA reports approved by A3 also need to be audited at A1 and A2. Therefore, D1 further implements ERC behavior towards A1 and A2. At the same time, as the office manager of the supervision section, A4 is responsible for the specific environmental regulation matters. As a result, A1, A2, and A3 further capture A4, eventually leading to the capture relationship spreading from A3 to the whole environmental regulatory authority.

### 4.2. Individual Network Analysis of ERC

#### 4.2.1. Centrality

**Figure 3:**
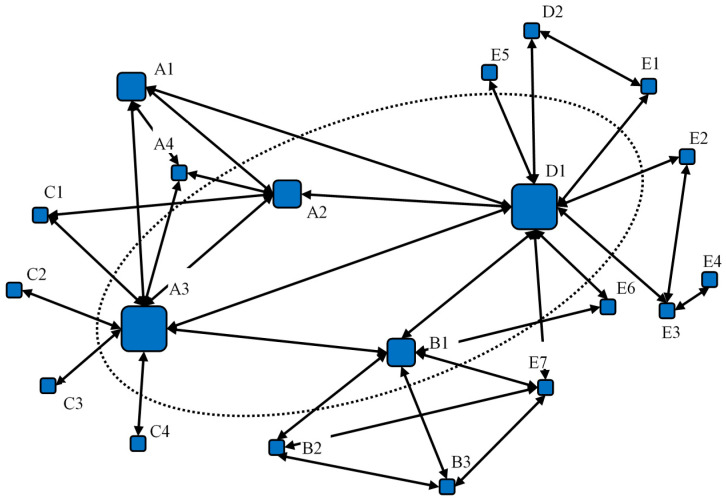
The core power circle of ERC network.

Table 2 depicts the measurement results of degree centrality, closeness centrality, and betweenness centrality for some dominant stakeholders in the ERC network. First, in terms of degree centrality, D1 has the highest degree centrality value, which indicates that D1 occupies the core position in the network and controls the greatest power to influence other stakeholders. Its ERC behaviors are the most frequent and serious. In addition, A3, B1, and A2 also have high degree centrality values. As illustrated in Figure 3, together with D1, they establish the ERC supreme power circle. The four stakeholders are the main actors in this case. Second, in terms of closeness centrality, D1, A3, B1, and A2 have the highest closeness centrality, and are the least controlled by other stakeholders, indicating that they are at the core of this relationship network. Third, in terms of betweenness centrality, the characteristics and trends presented are almost consistent with those of the first two centrality indicators. The degree centrality of E7 and E3 is lower, but their betweenness centrality is higher; even higher than that of A2. This indicates that though they cannot dominate other stakeholders in the network, they act as an intermediary to facilitate the communication and interaction among other actors. They are in an important position in this ERC case. With the help of its partnership with D1, E3—the business manager of XY company—helps the poultry farmer E4 to handle the business of EIA report-writing from HB company. Thus, a linkage between D1 and E4 is established, and the latter gets involved in this ERC network. Similarly, E7, the boss of a metallurgical plant in Bobai country, learns from B3 about HB’s EIA report-writing business. E7 then actively seeks out D1 for business cooperation and thus becomes the intermediary among D1, B2 and B3. By contrast, as the director of Bobai EPB, A1 owns the supreme authority in the implementation of environmental regulations; but A1′s passive captured behavior mainly aims towards cooperation with A3′s active capture behavior. A1 does not have an impetus for implementing ERC in practices. As such, A1 cannot exert a dominant influence on other stakeholders’ actions. A1 contributes to this ERC incident by acquiescing in the illegal acts of A2 and A3.

According to the centrality analysis, it is demonstrated that the ERC case forms a core power circle consisting of D1, A3, B1, and A2 (as illustrated in Figure 2). The capture behavior and collusion interests mainly occur in the environmental regulatory authority and the agency. D1 is the general manager of HB company whose major business is EIA report-writing, and D1′s work is to canvass business orders and dock with the regulatory authority. He owns the broadest linkages in this incident. An interest pyramid of “total contract-subcontract” is formed by him. Therefore, we consider him to be the primary person responsible for this case, i.e., the initiator. Besides, B1, A2, and A3 are the principal leaders of the commissioned regulatory agency and environmental regulatory authority, respectively. They have the final say in the review and approval of EIA reports and the supervision of corporate operations. Meanwhile, they not only have the need for passive capture, but also have the motivation for initiative rent-seeking. For example, A3 contacts D1 on his own initiative after receiving the EIA report from DX hotel (writing by HB company), and implies that only by paying the “approval fee” will this report be approved. Therefore, we consider A3, B1, and A2 to be the main persons responsible for this ERC case. Figure 3 also illustrates that D1, A3, B1, and A2 have a large number of relationships and extensive linkages, so they hold absolute control over the ERC network.

#### 4.2.2. Cohesive Subgroups

**Figure 4:**
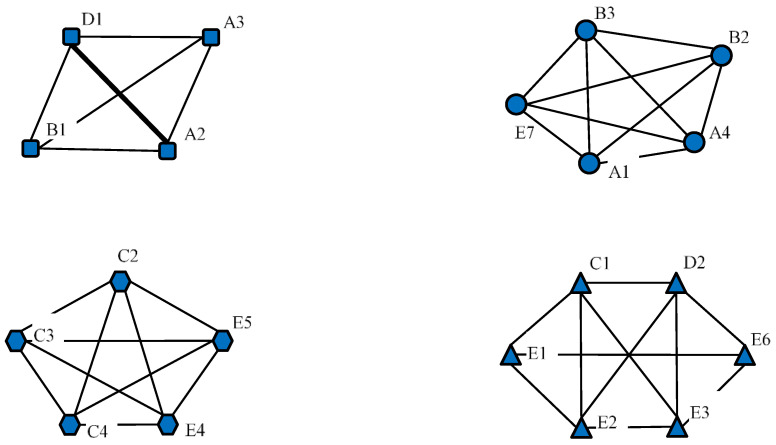
The classification and composition of ERC cohesive subgroups.

Furthermore, we use the imported relation matrix as the basis for subgroup analysis. In detail, four cohesive subgroups based on fitness are obtained with the help of the “K-cores” function of NetDraw 2. Figure 4 illustrates the stakeholder composition of four cohesive subgroups. The thickness of a line describes the degree of linkages between two stakeholders. It can be seen that D1, A3, B1, and A2 constitute a stable ERC circle. Their interactions are of great vitality and coherence. The capture behavior and interest transmission mainly occur among the four stakeholders. Besides, the capture behavior between D1 and A2 is calculated to be the most frequent. The twos dominate this ERC case.

Then, the “core—periphery” structure of this capture network is explored by using the “Continuous” function. After 1000 iterations, the correlation coefficient is up to 0.547, the mean of all stakeholders’ coreness values is equal to 0.179, and the standard deviation is equal to 0.134. From Table 3, the coreness ranking of D1, A3, A2, and B1 is still in the top four. This is consistent with the results of the subgroups analysis based on fitness partition. Therefore, we believe that these fours are core structure subjects, while the remaining sixteens are on the periphery of this ERC network.

D1 is the first initiator of ERC behavior in reality. He owns the most frequent relationships and thus possesses great information advantages and rich social resources. On the one hand, with the growth of HB’s business volume, D1 is increasingly occupying the core position of this capture network. On the other hand, the interest transmissions among stakeholders are becoming more frequent, and former customers are constantly bringing new businesses to HB company. For example, like E7, E6—the boss of a wood processing factory in Bobai country—learns from B1 about HB’s EIA report-writing business and eventually purchases the business from D1. As a result, D1′s core role in the network is constantly consolidated, which further enhances the linkages with A3, B1, and A2, and promotes the establishment of a core power circle. As such, members of the core power circle have the motivation to implement ERC behavior proactively. D1 is the initiator in this case, while A3, B1, and A2 are the main responsible persons.

Other stakeholders are defined as periphery structure nodes in the network. They are dependent on the core structure subjects, and their motive is to provide help for the core subject’s capture behavior, and gain benefits. For example, although A1 has no direct linkage with the supervision bureau, HB company or enterprises, he has extensive linkages and resource mobilization capabilities within the environmental authority. He acquiesces in the bribery of A2, A3, and A4 with rent-drawing, thus helping D1 realize ERC and gaining huge benefits (A1 in this case ultimately gets the highest illegal income owing to his leadership). In addition, as essential partners of the core subjects, A4, B2, B3, and D2 either help to handle specific matters, selectively supervise the illegal emission of enterprises, or drum up deals for the EIA report-writing business. They provide auxiliary support for core subjects, and also obtain a small amount of benefits. Therefore, we believe that periphery structure subjects have no higher motivation to break or flee the network, and they are the indirect responsible persons in this case.

#### 4.2.3. The Structural Hole

Table 4 depicts the measurement value of the structural hole index of the main stakeholders in the ERC network. D1 and A3 have the largest effective scale value and efficiency value among all stakeholders. This indicates their capture behaviors are unrestricted and efficient. Besides, their constraint values are both the smallest. Thus, D1 and A3 are located in the position of structural holes and can easily apply the control advantage to influence other actors’ behaviors. Surprisingly, A3 has a higher hierarchy value than D1. It is demonstrated that A3 is located at the core of this core power circle. In fact, A3, as a spokesperson of the environmental regulatory authority, not only captures superior leaders to engage them in collusion, but also opens up the channel for enterprises to communicate with the supervision bureau. In addition, A3 buys off the EIA experts and manages the jury meeting. He plays the role of “bridge” to promote exchanges and cooperation, thus possesses the most prominent centrality.

Although B1, the head of the supervision bureau, and A2, deputy director of the Bobai EPB, are also at the core of this network, they are not capable of initiating ERC actively. As shown in Table 5 by the honest broker measurement, B1 and A2 have the largest number of intermediary sizes and pairs except D1 and A3, thus illustrating that the main purpose of their behavior is to cooperate with interactions between D1 and A3. Therefore, even though B1 and A2 have high effective scale values and efficiency values, and they are also located in the core power circle, they cannot control the exchanges of information or interests substantially like D1 or A3. B1 and A2 are essential intermediaries in the network. They make full use of their extensive relationships to play the role of intermediary and profit from it. For example, taking advantage of his leadership role, B1 requires personnel at the supervision bureau to selectively supervise the illegal emission of polluting enterprises, thereby helping D1 capture environmental regulations implemented by the supervision bureau. However, the cost is that D1 must pay a “supervision fee” of CNY 1000 per EIA report.

## 5. Discussion

Drawing on the regulatory capture and principal—agent theories, this study explored actor roles, interest transmission relationships, behavior motives, and mechanisms of ERC. A case study based on multisource matched data revealed that ERC has the characteristics of concealment and complexity. The environmental regulatory authority (e.g., A2 and A3), commissioned regulatory agency (e.g., B1), and agency of enterprises (e.g., D1) form the core power circle of the ERC network, in which the first two play the role of intermediary and the latter acts as an initiator. In addition, they occupy the core position of the ERC network and have the motivation to proactively implement ERC behavior. Periphery structure stakeholders are dependent on the core structure actors, and their motive is to provide assistance to the core actors’ capture behavior and thus gain benefits.

### 5.1. Theoretical Contributions

The current research makes several crucial theoretical contributions to the relevant literature. First, we apply the regulatory capture theory to the field of environment-related research, making up for the lack of past literature focusing on regulatory capture in the economic field [19,43]. The regulatory capture incident concerning residents’ livelihood (e.g., the environment) is widespread [44,45], but the economic development often comes first, especially in emerging economies or in the face of major crises [46]. As a result, we rarely explore regulatory capture outside the economic sphere. The discussion of ERC in this article contributes to an improved framework of regulatory capture research. Meanwhile, different from the macrolevel perspective commonly adopted in the existing research, we conduct a thorough analysis of the typical ERC case to illustrate the mechanism of the ERC incident. Our research reveals the network roles of stakeholders and their motives for participating in ERC, thus providing targeted implications for designing practical preventive measures and valuable suggestions for evading other types of regulatory capture.

Second, the study combines the practices of China’s environmental regulations to extract a triple-layer principal—agent model, which adds to a growing understanding of actor roles and behavior interactions in ERC. Although the classic principal—agent model explains the potential causes and mechanisms by which the commissioned regulatory agency captures the environmental regulatory authority [8], the concealment and complexity characteristics of ERC behavior are omitted. Especially in grassroots authority, to increase their own interests, the environmental regulatory authority has the will to actively seek rent and deceive the superior government [47]. The agency of enterprises naturally possesses the motive of active capture. Therefore, the interest transmission network has been constantly strengthened, and core actors’ roles and positions have also been continuously consolidated. Based on a typical ERC case, the study identifies the interactions among multiple stakeholders and illustrates the complex and hidden relations in ERC. Our research thus develops the principal—agent model.

Third, although the literature has investigated the regulatory capture issue using different quantitative methods, such as field studies and modeling stimulation [34,48], our research is an initial attempt to integrate the quantitative method (i.e., the SNA approach) and qualitative analysis (i.e., the case study) to uncover the process by which an individual’s interest transmission behavior evolves into the collective ERC. ERC involves multiple stakeholders. The interests of different sectors are intertwined. Interactions between individuals are hidden behind formal business dealings. It is difficult to elaborate on the real motives and deep-seated causes by analyzing stakeholders’ behavior from the theoretical perspective. Our analysis of a specific ERC case explains the interactions between stakeholders, restoring the evolution process of ERC in real situations, and enhancing the credibility of research conclusions. Besides this, we also use the SNA approach to quantify the network role of each actor and its structural position, making the relationships of ERC visible. The effort of integrating qualitative and quantitative methods provides a methodological reference for future research.

### 5.2. Practical Implications

The findings also have important implications for management in environmental regulations. First, the ERC presents an obvious characteristic of collective collusion. Individual capture often evolves into collective capture. Network members will gradually lose the motivations and abilities to break or flee the relationship network, along with the constant consolidation of interest networks and frequent interactions among stakeholders. Thus, the principle of flattening must be considered when constructing an organizational structure, in order to realize the decentralization and balance of power, thereby weakening a single subject’s control of collective actions. It is necessary to promote the transformation of the environmental regulation principal–agent mode from a peer or superior government (as the single supervisory entity) to multiple subjects. The command-and-control regulatory mode should be supplemented by the synergetic effects of the public, news media, industry associations, and other multiple subjects so as to establish a long-term mechanism of ERC governance [49].

Second, the SNA results show that different stakeholders are in different positions and hold different degrees of control in the network. Both the enterprises (the role represented by D1) and the environmental regulatory authority (the role represented by A3) are at the core of this capture network, and occupy the position of structural holes. They are the initiator and intermediary for ERC incidents, respectively. Thus, it is suggested that quantitative methods, such as the SNA approach, should be applied to clarify the accountability of the primary responsible persons, the main responsible persons, and the indirect responsible persons for quantifying stakeholders’ responsibility in the final punishment. The peer or superior government departments should intensify the fight against collusion and interest transmission between officials and businesses, so that the cost of ERC greatly exceeds its benefits. The huge penalties for accident enterprises and administrative accountability for regulatory authorities have been demonstrated to be valid in reducing the willingness of peripheral structure subjects to participate in the ERC [31].

Third, the ERC is dominated by core structure subjects (e.g., D1, the agency of enterprises), but they still need intermediaries (e.g., E3, the business manager of XY company) to play the role of “bridge”. An intermediary is dependent on core structure subjects, so the core structure subject can effectively achieve its capture purpose by the help of intermediaries’ extensive relations. Therefore, breaking the interest community is key in order to evade ERC. It is necessary to separate the rights of jurisdiction and supervision in order to encourage more stakeholders to participate in environmental regulations and report illegal acts. The government can establish an exchange system for environmental-regulatory personnel to carry out regulations across regions. Especially when the ERC happens, local environmental regulators must not participate in the investigation process. Instead, environmental regulators should be dispatched from other regions, so as to prevent regulators from establishing an interest transmission relationship with the regulated enterprises during long-term contacts.

Finally, there is not necessarily a direct relationship between interest subjects in the peripheral position, but once they play the role of intermediary, they will draw more conspirators over to this network, thereby expanding the network scale and profiting from it. Therefore, a blacklist system can be established in the field of environmental protection to regulate the professional and social morality of all parties and induce a fear of ERC among enterprises and authorities. The labor union system has been proven to effectively restrain individual behavior all over the world [50]. As a result, the government should encourage the establishment of the labor union within the enterprise so as to increase employees’ recognition of the collective and enhance their enthusiasm in participating in production and supervision. In addition, the whistleblower system can encourage employees to report illegal business activities, thus forming a bottom-up, internal supervision mechanism.

### 5.3. Limitations and Future Directions

Selecting a typical incident in the field of environmental regulations for case analysis, this study deeply analyzes the micro-mechanism for ERC, clarifies the main stakeholders and interest exchanges in this network, and bridges the gap between macro-phenomena analysis and micro-interaction analysis. Despite the implications of curbing regulatory capture problems, the current research has some limitations, which suggest meaningful future research directions. First, we focus on the ERC behavior from the perspective of interest transmission, but as social persons, stakeholders are affected by irrational facts. Thus, estimating the impact of individual psychological factors on ERC behavior may be one area where we can extend this study. Second, the actors and structure of the ERC network are constantly changing. Will the ERC network structure change significantly and regularly? Do the core structure subjects always occupy the structural hole position? Therefore, future researchers may explore the dynamic evolution of the ERC network. Additionally, we select a typical case to uncover the ERC process, which limits the generalizability of the research conclusions to some extent. It is recommended that future research conduct a comparative analysis of multiple cases to improve the credibility and validity of our conclusions.

## 6. Conclusions

Past regulatory capture studies generally take the macro-perspective of the state or industry and focus on the economic field. The current study adds to the regulatory capture literature by providing one typical ERC case, showing what role network actors/stakeholders play and how their behavior motives and interactions trigger the ERC incident. It is found that the ERC presents obvious characteristics of concealment and complexity. The environmental regulatory authority and commissioned regulatory agents play the role of intermediary, and the agents of the enterprises act as an initiator. They together dominate the evolution of the ERC network. Peripheral structure subjects play the role of “bridge” and aim at expanding the network scale and profiting from it. The study also graphically explicates linkages among stakeholders and the structure of the ERC network using the SNA approach. Integrating the theoretical analysis in a real ERC context, we not only reveal the hidden interactions and interest transmission mechanism of ERC, but also extend the analytical framework of regulatory capture and principal–agent theories. We hope this study can attract more attention to the regulatory capture issue and more fine-grained, dynamic, and comparative research on the evolution of ERC.

## Figures and Tables

**Table 1:** The coding List of Environmental Regulatory Capture (ERC) Network Stakeholders.

Organization	Subject	
Authority	A1 Director of Bobai EPB	A2 Deputy director of Bobai EPB
	A3 Deputy chief of supervision section	A4 Office manager of supervision section
Supervision bureau	B1 Head of the supervision bureau	B2 Deputy head of the supervision bureau
	B3 A key clerk	
Evaluation organization	C1 EIA expert 1	C2 EIA expert 2
	C2 EIA expert 3	C2 EIA expert 4
Agency	D1 General manager of HB company	D2 Vice general manager of HB company
Enterprise	E1 Boss of DX hotel	E2 Boss of XY company
	E3 Business manager of XY company	E4 Boss of a livestock farm
	E5 Boss of an automobile maintenance plant	E6 Boss of a wood processing factory
	E7 Boss of a metallurgical plant	

**Table 2:** The centrality of ERC Network.

Stakeholder	Degree Centrality	Closeness Centrality	Betweenness Centrality
D1	57.895	27.000	60.819
A3	47.368	30.000	40.058
B1	31.579	33.000	17.154
A2	26.316	36.000	4.678
A1	21.053	37.000	1.657
E7	21.053	40.000	4.873
E3	15.789	42.000	10.526
B2	15.789	49.000	0.000

**Table 3:** The “core—periphery” Structure Analysis of ERC Network.

Sort	Stakeholder	Coreness	Structure	Sort	Stakeholder	Coreness	Structure
1	D1	0.607	At the core	11	E1	0.128	On the periphery
2	A3	0.403		12	E2	0.128	
3	A2	0.295		13	C1	0.121	
4	B1	0.294		14	E5	0.105	
5	A1	0.273	On the periphery	15	B2	0.103	
6	E7	0.197		16	B3	0.103	
7	A4	0.171		17	C2	0.069	
8	E6	0.158		18	C3	0.069	

**Table 4:** Measurement Values of the Structural Hole.

	Effective Size	Efficiency	Constraint	Hierarchy
D1	8.833	0.736	0.302	0.198
A3	6.800	0.680	0.355	0.200
B1	3.751	0.510	0.463	0.039
A2	2.333	0.389	0.542	0.039
E3	2.000	0.500	0.704	0.057
E7	1.800	0.360	0.627	0.024
A1	1.400	0.280	0.638	0.008
B2	1.000	0.250	0.766	0.000

**Table 5:** Measurement values of the honest broker indices.

	Size	Pairs	HBI0	HBI2
D1	11	55	47	8
A3	9	36	29	7
B1	6	15	9	6
A2	5	10	4	6
A1	4	6	1	5
E7	4	6	2	4
E3	3	3	2	1
B2	3	3	0	3

Note: HBI0 means the Honest Broke Indicator 0, HBI2 means the Honest Broke Indicator 2.
